# Heartbeat-evoked potentials following voluntary hyperventilation in epilepsy patients: respiratory influences on cardiac interoception

**DOI:** 10.3389/fnins.2024.1391437

**Published:** 2024-07-05

**Authors:** Niovi A. Stoupi, Marieke L. Weijs, Lukas Imbach, Bigna Lenggenhager

**Affiliations:** ^1^Department of Psychology, University of Zurich, Zürich, Switzerland; ^2^Department of Neurology, University Hospital of Zurich, Zürich, Switzerland; ^3^Swiss Epilepsy Center, Klinik Lengg, Zürich, Switzerland; ^4^Zurich Neuroscience Center, University of Zurich and ETH Zurich, Zürich, Switzerland

**Keywords:** interoception, heartbeat-evoked potential, voluntary hyperventilation, epilepsy, respiration, autonomic regulation

## Abstract

**Introduction:**

Current evidence indicates a modulating role of respiratory processes in cardiac interoception, yet whether altered breathing patterns influence heartbeat-evoked potentials (HEP) remains inconclusive.

**Methods:**

Here, we examined the effects of voluntary hyperventilation (VH) as part of a clinical routine examination on scalp-recorded HEPs in epilepsy patients (*N* = 80).

**Results:**

Using cluster-based permutation analyses, HEP amplitudes were compared across pre-VH and post-VH conditions within young and elderly subgroups, as well as for the total sample. No differences in the HEP were detected for younger participants or across the full sample, while an increased late HEP during pre-VH compared to post-VH was fond in the senior group, denoting decreased cardiac interoceptive processing after hyperventilation.

**Discussion:**

The present study, thus, provides initial evidence of breathing-related HEP modulations in elderly epilepsy patients, emphasizing the potential of HEP as an interoceptive neural marker that could partially extend to the representation of pulmonary signaling. We speculate that aberrant CO_2_-chemosensing, coupled with disturbances in autonomic regulation, might constitute the underlying pathophysiological mechanism behind the obtained effect. Available databases involving patient records of routine VH assessment may constitute a valuable asset in disentangling the interplay of cardiac and ventilatory interoceptive information in various patient groups, providing thorough clinical data to parse, as well as increased statistical power and estimates of effects with higher precision through large-scale studies.

## Heartbeat-evoked potentials following voluntary hyperventilation in epilepsy patients: respiratory influences on cardiac interoception

The mind and body are inevitably intertwined. Current research increasingly targets interoception (i.e., the sense of the internal bodily state; Craig, 2002) as an indispensable source of sensory input that significantly influences mental processes (Azzalini et al., 2019). The heartbeat-evoked potential [HEP, (Schandry et al., 1986)] is regarded as an electrophysiological index of the cortical processing of cardiac interoceptive information (Park and Blanke, 2019). Higher HEP amplitudes have been found when participants focus on interoceptive states (Petzschner et al., 2019). At the same time, altered HEPs have been observed during multiple mental processes (Schulz et al., 2013; Luft and Bhattacharya, 2015; Sel et al., 2017; Park et al., 2018; Richter and Ibáñez, 2021) as well as in clinical conditions (Terhaar et al., 2012; Schulz et al., 2015; Wei et al., 2016; Lutz et al., 2019; Pang et al., 2019). Importantly, although the exact nature of the interplay between HEPs and cardiac dynamics is still unclear (Coll et al., 2021), changes in HEPs seem related to cardiac interoception rather than variation in cardiac signaling (Park and Blanke, 2019).

While HEP is the most used neural indicator of interoception and has often been investigated in isolation, the brain receives ascending signals from diverse inner organs (Critchley and Harrison, 2013). Breathing, for example, generates an abundance of ascending signals (Heck et al., 2017), associated with modulatory effects on other interoceptive functions, including cardiac processing (Critchley and Harrison, 2013). Indeed, the respiratory and cardiovascular systems are firmly coupled (Berntson et al., 1993; Ben-Tal et al., 2012; Dick et al., 2014a). Surprisingly, though, knowledge regarding the interplay between cardiac and respiratory interoceptive processing is scarce. In a behavioral study (Garfinkel et al., 2016), for instance, individual interoceptive awareness, referring to metacognitive insight into one’s ability to accurately detect internal bodily signals (Garfinkel et al., 2015), was significantly correlated across the heart and breathing dimensions, hinting at partially overlapping mental representations. This is supported by brain imaging studies (Hassanpour et al., 2016, 2018), highlighting a common cortical substrate, the right mid-insula, in the representation of sympathetically-induced changes in cardiorespiratory states. Integration of cardiorespiratory afferents at the cortical level could encode the physiological arousal, involving cardiorespiratory coupling by default (Ben-Tal et al., 2012; Dick et al., 2014a), that necessitate an attentional shift toward interoceptive signaling to initiate adaptive behavior (Critchley and Harrison, 2013) and possibly also related to emotional awareness (Critchley and Garfinkel, 2017) and decision making (Herman and Tsakiris, 2021). Electroencephalography (EEG) studies (Immanuel et al., 2014; Baumert et al., 2015) showing altered HEP waveforms during sleep in children with sleep-disordered breathing establishing a pathophysiological link between disrupted respiratory patterns and cardiac neural processing in populations with cardiopulmonary dysfunctions. Importantly, though, these studies have included solely sleep periods free from abnormal respiratory events in their analysis. Thus, the question of whether altered breathing patterns can affect the neural processing of cardiac information arises.

As far as the authors are aware, only one study (MacKinnon et al., 2013) has investigated how changes in ventilatory pattern relate to cardiac interoception by evaluating the effects of resonant breathing (i.e., slow diaphragmatic respiration) on HEPs in healthy participants. Specifically, the authors found an increased HEP amplitude during resonant breathing compared to baseline in one out of three recording EEG channels, providing preliminary evidence on the modulatory role of respiratory signals in HEP activation. This could expand the existing understanding of cortical mechanisms underlying breathing perceptions (von Leupoldt et al., 2010a,b, 2011; Chenivesse et al., 2014), as well as aid in elucidating the relationship between respiration and higher-order mental functioning, such as bodily self-consiousness (Adler et al., 2014; Allard et al., 2017) and facial recognition (Vinckier et al., 2018). It is also noteworthy that many neurological conditions (Cragg et al., 2015; Chen et al., 2017; Racca et al., 2020) are associated with disrupted cardiorespiratory processes. Thus, a cardiac interoceptive neural marker relating to breathing might be of considerable clinical relevance in interpreting possible interoceptive irregularities in such pathophysiological conditions.

Epilepsy is a neurological disorder linked to altered patterns of both cardiac and ventilatory activity (Moseley and Britton, 2014; Myers et al., 2018; Druschky et al., 2020), which is often encountered in HEP literature due to methodological considerations (Kern et al., 2013; Babo-Rebelo et al., 2016; García-Cordero et al., 2017; Park et al., 2018). A common activation procedure in clinical settings for the diagnosis and classification of seizure disorders is voluntary hyperventilation (VH), involving distinct electrographic responses associated with the physiological mechanisms inherent to VH (Godoy et al., 2017; Rana et al., 2023). Specifically, VH is related to EEG morphologic changes with brain wave activation manifested as a buildup of delta and theta activity (Siddiqui et al., 2011; Acharya and Acharya, 2021), a phenomenon observed also in non-epileptic individuals (Salvati and Beenhakker, 2019). At the same time, VH may trigger epileptiform discharges and clinical seizures in an appreciable portion of susceptible patients (Rana et al., 2023), although this seems to be an infrequent, as well as an epilepsy-specific, occurrence (Holmes et al., 2004; Kane et al., 2014; Alghamdi et al., 2021) that also appears to alter with increasing age (Marsden et al., 2012; Ahdab and Riachi, 2014). The processes contributing to VH-induced EEG changes remain unclear (Rana et al., 2023), with several studies suggesting a multifactorial combination of numerous concomitant physiological changes following VH (Son et al., 2012; Assenza et al., 2015; Kanaan et al., 2022; Wennberg, 2022; Rana et al., 2023), also introducing the crosstalk between cortical and subcortical structures that may involve interoceptive components and their response to altered breathing patterns (Salvati and Beenhakker, 2019; Salvati et al., 2022). Thereby, exploring cardiac interoceptive indicators and their alterations in epileptic patients during VH could aid in better understanding the disorder, but also interoceptive communication in general.

Here, we investigated how VH affects HEPs in epilepsy. Appertaining to “proof of concept” research, HEPs were derived from already recorded EEG scalp data of epilepsy patients, who have previously undergone routine EEG (rEEG) assessment with VH. VH constitutes a standard activation protocol in neurodiagnostic settings (Sinha et al., 2016) and, therefore, any obtained effects could be validated in subsequent studies with larger samples by retrospectively reviewing patient records and relating findings to specific symptoms. Following a quasi-experimental methodology, we compared HEP amplitudes across pre-VH and post-VH conditions in a sample of young and elderly adults. As VH-induced changes in breathing patterns should call for heightened attention to interoceptive afferents (von Leupoldt et al., 2010a; Petzschner et al., 2019), the main hypothesis was that HEP amplitudes will be significantly increased following the VH intervention, as compared to baseline. To the knowledge of the authors, this is the first study examining the effects of VH on HEPs.

Next to the principal research question, the current work also investigated whether VH effects on HEP waveforms differ as a function of age and sex. The motivation for such analysis was based on previous studies suggesting differences in interoceptive processing as a function of age (Khalsa et al., 2009; Murphy et al., 2018) and sex (Grabauskaitė et al., 2017). With regards to age, it was expected that young subjects will exhibit overall greater HEP waveforms in comparison to elderly participants, as interoceptive capability is thought to decrease with age (Murphy et al., 2018; but see Kamp et al., 2021). It was, furthermore, anticipated that age will interact with VH condition, with HEP amplitude being higher following VH, compared to baseline, and this effect being stronger for younger participants, thus denoting an increased interoceptive regulation for young adults (Khalsa et al., 2009; Murphy et al., 2018), in comparison to older individuals. Concerning sex, on the other hand, it was speculated that females will generally show greater HEP amplitudes, in comparison to males, as female participants display overall heightened interoceptive awareness (Grabauskaitė et al., 2017) and have also previously demonstrated higher HEPs compared to men (MacKinnon et al., 2013), however, no interaction effect was expected.

## Materials and methods

### Participants

A total of 80 epilepsy individuals (M = 50.5, SD = 20.77) were selected from the University Hospital of Zurich (USZ) patient database, prioritizing the most recent system entries. Half of the included EEG data pertained to young participants (M = 30.8, SD = 7.79), while the other half involved elderly individuals (M = 70.2, SD = 4.17). The number of female and male participants was counterbalanced in both age subgroups. As the research topic of the study is novel, no information exists in the literature regarding effect sizes or variance components, so as to conduct a power analysis (Larson and Carbine, 2017). Accordingly, target participant number was chosen a-priori based on the sample size of other HEP studies, which overall ranges from 5 to 50 participants, with a mean of 21.80 participants per experimental group (Coll et al., 2021).

**FIGURE 1:**
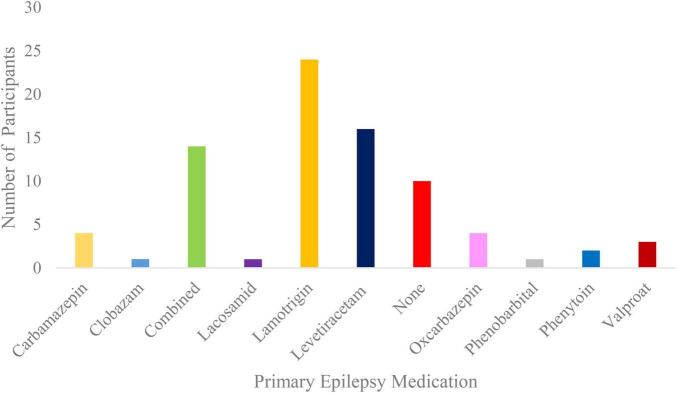
Amount of primary epilepsy medication used across participants.

All participants had the option to stop VH at any time. Solely data from patients that underwent the whole VH procedure were selected for this study. Inclusion criteria involved an age range of 18 to 45 years for young adults and of 65 years of age and above for older adults, as well as an overarching epilepsy diagnosis without differentiating between epilepsy subtypes. The rationale for not including middle-aged patients concerned the primary interest of the overarching study in comparing VH-induced effects on HEPs within the young and elderly adult groups, whilst using an age-group design similar to other studies on heartbeat-related signals (Kamp et al., 2021; López Pérez et al., 2023), thus enhancing comparability. Concerning epilepsy diagnosis, 35% of the cases consisted of generalized onset epilepsy, 31% of focal onset epilepsy, 1% of combined generalized and focal onset epilepsy and 33% of unknown onset epilepsy. Mean age of onset was 37.90 years with a standard deviation of 21.40 years. Primary epilepsy medication of the participants is presented on Figure 1.

Electroencephalography data entailing technologist reports of multiple electrode corrections and displacements, as well as of multiple pulse artifacts or overall abnormal cardiac activity, were excluded from the sampling process. Other exclusion criteria involved any type of epileptic activity, including interictal spikes, during the EEG recording, reported cardiovascular disease (e.g., coronary heart disease, atrial fibrillation) and installed cardiac pacemakers. Moreover, if the time period before, during and following VH did not reach a duration of 3 min, the recording was excluded. Lastly, participants were not included in the sample, if the hyperventilation intervention exceeded by far the 3-min time period, overlapped with the photic stimulation intervention, or if hyperventilation performance was evaluated as poor by the EEG technician. All participants provided informed consent. The study has been approved by the Cantonal Ethics Committee Zurich (approval number: 2019-00890) and was preregistered at the Open Science Framework (OSF; Soderberg, 2018). The OSF registry entry is accessible via https://osf.io/2hz6a/.

### Materials

Raw EEG data were acquired with a Nihon Kohden Neurofax EEG-1100 Unit (Nihon Kohden Corporation, 2015), using silver-silver chloride (Ag/AgCl) EEG Bridge Electrodes and following the routine international 10/20 scalp electrode placement system (Acharya et al., 2016). Anterior temporal T9/T10 electrodes were also added, resulting in a total of 23 EEG electrodes, with C3 and C4 being the online reference electrodes. All electrode impedances were below 5 kΩ. Cardiac monitoring was effectuated via a three-channel electrocardiogram (ECG) with standard limb leads according to the Einthoven’s triangle configuration. The sampling rate for all measurements was 200 Hz. Importantly, lower sampling frequencies appear to not significantly impact spectral and baroreflex parameters, including HRV (Ellis et al., 2015), even for pathologically decreased variability of RR intervals in patients (Ziemssen et al., 2008).

### Procedure

Electroencephalography electrodes were positioned on the participants’ head for the rEEG and signal quality was verified visually for movement or electrode artifacts. Participants were then asked to keep their eyes closed throughout the VH protocol, unless instructed otherwise. Subsequently, resting-state electrophysiological activity was recorded for 3 min. During that period, an activation procedure requiring participants to shortly open and close their eyes also took place, according to standard clinical EEG practice (Sinha et al., 2016). The VH intervention followed shortly afterward, unless indicated otherwise by medical (e.g., sickle cell disease or trait) or justifiable (e.g., participant inability or unwillingness to cooperate) reasons. Patients were instructed to breathe deeper and faster than normal with maximal effort in room air for 3 min. The recording continued for 3 min succeeding VH cessation. Participants were seated for the whole process. ECG was recorded contemporaneously throughout the rEEG.

### Design

This retrospective cohort study followed a mixed-subjects design. The within-subjects factor referred to the VH intervention, with a pre-post comparison of measured variables. On the between-subjects level, age (young or elderly) and sex (male or female), acquired from screening participant information during the data selection process, comprised the grouping variables. HEP amplitude (measured in μV) constituted the dependent variable of interest, obtained by averaging EEG data time-locked to the R-peak of the accompanying ECG signal. Cardiac variables for control analyses were derived from the ECG recording and included heart rate (HR; measured in bpm), heart rate variability (HRV; measured in %), root mean square of successive differences (RMSSD; measured in ms), as well as mean ECG amplitude time-locked to the R-peak (mECG; measured in μV). HRV was calculated according to a geometric method based on relative RR intervals, defined as the difference of consecutive RR intervals weighted by their mean, which are then mapped in a return plot with the median distance to the center point used to assess HRV (Vollmer, 2015).

### Data preprocessing

Recordings were analyzed using EEGLAB v2019.1 (Delorme and Makeig, 2004), an interactive MATLAB (Moler and Little, 1984) toolbox for electrophysiological data processing. Using an EEGLAB plug-in extension, called HEPLAB (Perakakis, 2019), ECG data were initially filtered with an embedded 2nd order Butterworth filter with a low cut-off frequency of 3 Hz and a high cut-off frequency of 30 Hz to facilitate R-peak detection. R-peak events were then identified via a built-in slow R-peak detection algorithm (de Carvalho et al., 2002), whilst R-peak detection was also verified visually. HR, HRV, and RMSSD measures were obtained using HRVTool 1.07 (Vollmer, 2015, 2019), an open-source MATLAB toolbox for HRV analysis.

EEG recordings were first filtered offline using a Notch filter at 50 Hz, corresponding to the frequency of alternating current in Europe. An additional band-pass filter was applied with a low-pass filter cut-off frequency of 30 Hz and a high-pass filter cut-off frequency of 0.16 Hz to minimize filter-induced distortions (Widmann and Schröger, 2012; Widmann et al., 2015). Apropos of a preliminary analysis for the development of an automated EEG data processing algorithm, only data pertaining to young participants (*N* = 40) were preprocessed manually hereafter. This involved a visual inspection of the EEG recordings for bad channels, as well as an Independent Component Analysis (ICA) in order to remove ocular artifacts, such as eye movements and blinks (Chaumon et al., 2015). Subsequently, any detected bad electrodes were interpolated using an inbuilt spherical spline algorithm (Perrin et al., 1989).

The young participant recordings were also preprocessed with Automagic (Pedroni et al., 2019), a MATLAB-based toolbox for EEG data processing compatible with EEGLAB. The findings ensuing from the manual and the automatized analysis were compared statistically to identify the ideal Automagic settings for the processing of the entire study dataset. These involved the second and third bad channel identification algorithms of the EEGLAB plugin “clean_rawdata(),” as well as an ICA-based artifact correction method, called ICLabel (Pion-Tonachini et al., 2019). The bad channel identification algorithms were set to detect channels with a lower correlation than 0.80 to a robust estimate based on other channels, whilst also using the default *z*-estimate settings (i.e., 4 standard deviations above channel population mean) for excluding EEG channels with excessive line noise. ICLabel extracted ICA components with a detection threshold of 0.8 for eye and muscle artifacts, after temporarily high-pass filtering the data at 2 Hz (Winkler et al., 2015). Automagic, then, employed a spherical spline algorithm for the interpolation of bad channels. The automated software was thereafter employed for the analysis of the entire participant data (*N* = 80), with the aim to ensure objectivity in HEP analysis and eliminate potential sources of result variability (Park and Blanke, 2019), as well as automatize the preprocessing of EEG datasets for prospective large-scale studies.

In all cases, after interpolation, data were re-referenced by means of the average reference, which is recommended for studying heart-brain interactions (Candia-Rivera et al., 2020) given its inherent independence with regards to specific head areas (Kayser and Tenke, 2010; Yao et al., 2019). As a final preprocessing step, EEG data were then epoched with the R-peak event as temporal reference. A baseline correction was not employed in order to avoid epoch artifacts from preceding cardiac field artifact (CFA) components, such as the P and Q waves from prior heartbeats (Petzschner et al., 2019). Epoch length ranged from −100 to 700 ms after the R-peak. Epochs involving extreme values, namely exceeding 75 μV in any of the channels, were rejected. If the proportion of rejected epochs exceeded 25% of the original epoch number in any VH condition, the participant was not included in the respective analysis (Petzschner et al., 2019). Epochs containing more than one R-peak were also excluded in order to ensure that any HEP effects observed did not result from the CFA of the succeeding heartbeat (Dirlich et al., 1997). If the remaining epochs exceeded 100, the participant was included in the corresponding analysis. Included epochs were then averaged within participants separately for each VH condition. For cluster-based HEP analyses, time window of interest (TOI) was set from 200 to 650 ms, roughly corresponding to the earliest (MacKinnon et al., 2013; García-Cordero et al., 2017; Pfeiffer and De Lucia, 2017) and latest (Schulz et al., 2013, 2015) time points in which HEP modulations have been formerly observed, without overlapping with the CFA of the next heartbeat (Petzschner et al., 2019). Regarding control analyses, mECG for included epochs was averaged within the TOI in order to examine whether ECG amplitudes over the specific time range could be confounded with the HEPs (Park and Blanke, 2019).

For statistical analyses, cluster permutation tests (Maris and Oostenveld, 2007) were performed in Fieldtrip (Oostenveld et al., 2011), an open-source MATLAB toolbox for analyzing EEG data. In order to maintain the statistical properties of the cluster-based test, we applied the *t*-test statistic (Maris and Oostenveld, 2007), since the sum of the statistic values across the cluster, known as cluster mass, compromises the mathematical equivalence of the *F*-values to *t*-test versions (Bullmore et al., 1999; Maris and Oostenveld, 2007; Oostenveld et al., 2011). We opted for the *t*-value on the within- and between-subjects level in order to maintain comparability to previous studies, given that cluster-based permutation testing has originally been developed for use with *t*-values (Wheldon et al., 2007; Oostenveld et al., 2011). Accordingly, the permutation distributions were derived from *t*-value statistics, based on 1,000 permutations to correct for multiplicity of tests (Winkler et al., 2016). There were on average 5.6 neighbors per electrode. A two-tailed Monte-Carlo *p*-value of 0.05 (uncorrected) was used as the initial cluster-forming threshold. Clusters were then marked as significant based on a critical test-wise alpha-level of *p* < 0.025, corresponding to a false alarm rate of 0.05 in a two-sided test (Maris and Oostenveld, 2007; Groppe et al., 2011). For all other statistical procedures, the standard threshold of *p* < 0.05 was used to establish significance. If the assumption of normality was violated, the corresponding non-parametric alternative was employed for statistical testing (Yan et al., 2017).

## Results

### Epoch number

Within- and between-groups comparisons did not reveal any significant differences in the number of included epochs (see Supplementary Table A1).

### Pre-VH/Post-VH comparisons

#### HEP

**FIGURE 2:**
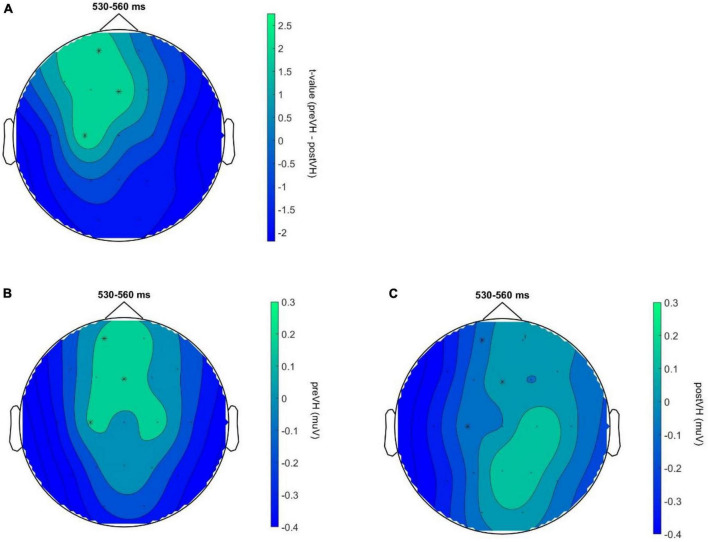
HEP topography for elderly participants across VH conditions. Neural responses to heartbeats for elderly participants (*N* = 40) across VH conditions. **(A)** Topography of the identified positive cluster (*p* = 0.006) depicting the *t*-values for the VH-level comparison during the time window of significant activation (530–560 ms). HEP differences at electrodes Fp1, C3 and Fz, indicated by an asterisk, remained significant throughout the entire cluster time-window (*p* < 0.01 at all highlighted electrode sites). **(B)** HEP topography for pre-VH showing mean HEP amplitude, averaged across the TOI, during the cluster time window with electrodes Fp1, C3 and Fz highlighted. **(C)** HEP topography for post-VH showing mean HEP amplitude, averaged across the TOI, during the cluster time window with electrodes Fp1, C3 and Fz highlighted.

**FIGURE 3:**
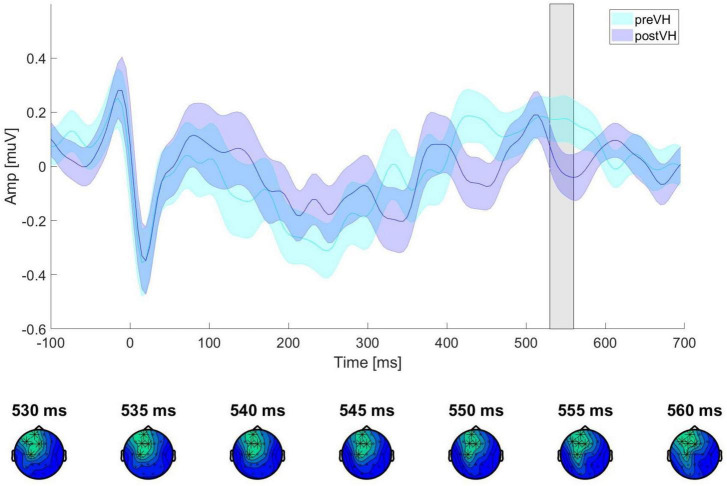
ERP waveforms for elderly participants across VH conditions. ERP waveforms showing HEP amplitude and standard error of the mean, averaged across all cluster electrodes (Fp1, F3, C3, F7, Fz) for pre-VH and post-VH conditions. The gray shaded area represents the time period (530–560 ms) in which a significant positive electrode cluster was identified (see Figure 2A). The topography below shows the electrodes contributing to the cluster at different time points (*p* < 0.01 at all highlighted electrode sites) during the time window of significant activation (530–560 ms).

In the total sample (*n* = 76), a cluster-based permutation analysis identified a positive cluster (*p* = 0.043) for the VH-level comparison, ranging from 525 to 545 ms and distributed over frontocentral sites. However, the cluster did not survive test-wise alpha-level correction. No significant cluster differences were found across pre-VH and post-VH for either the manually (all cluster-level *p* > 0.14) or the automatedly processed data (all cluster-level *p* > 0.35) in the young participant subgroup (*n* = 36). In the elderly group (*n* = 40), cluster-based permutation analyses indicated an effect of condition (*p* = 0.006), corresponding to a positive cluster from 530 to 560 ms, mainly distributed over frontal electrodes (see Figure 2), with maximum positive HEP difference observed at 550 ms after the R-peak (electrode C3), *t*(39) = 3.12, *p* = 0.006. Mean HEP amplitude averaged across the cluster was significantly higher during pre-VH compared to post-VH, *t*(39) = 3.91, *p* < 0.001, *d* = 0.62 (see Figure 3).

#### Control analyses

**FIGURE 4:**
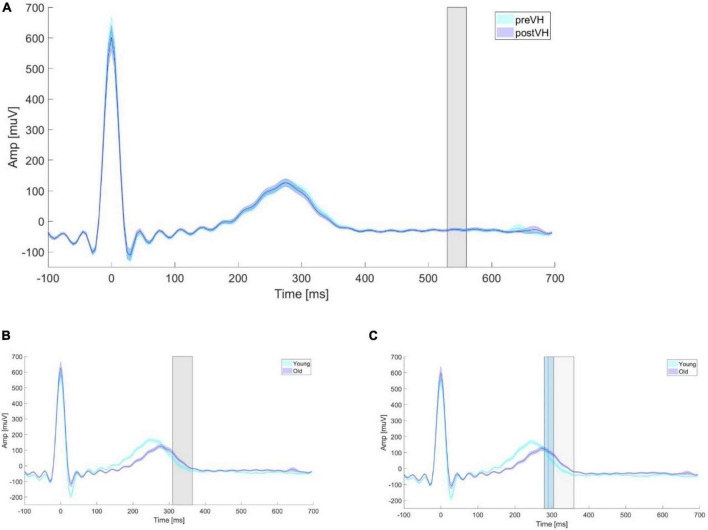
Mean ECG amplitude according to age and VH condition. Mean ECG amplitude averaged for age groups and VH conditions. **(A)** Mean ECG amplitude averaged for each elderly participant within pre-VH and post-VH across epoch length. The gray shaded rectangle depicts the time period (530–560 ms) in which a significant positive electrode cluster was identified in the EEG (see Figure 2A). **(B)** Mean ECG amplitude averaged for each young and elderly participant within pre-VH across epoch length. The gray shaded rectangle depicts the time period (310–365 ms) in which a significant positive electrode cluster was identified in the EEG (see Supplementary Figure A1). **(C)** Mean ECG amplitude averaged for each young and elderly participant within post-VH across epoch length. The gray and light-blue shaded rectangles depict the time period in which a significant HEP difference was identified in the EEG for the positive (290–360 ms) and negative (280–305 ms) clusters, respectively (see Supplementary Figure A3).

Within-subjects comparisons of heart parameters are summarized in Table 1. Within the TOI, mECG was not significantly different across conditions only for the elderly subgroup. Further analysis showed that, for the aforesaid group, mECG did not differ significantly across pre-VH (Mdn = −24.7, IQR = −41.9 to −10.5) and post-VH (Mdn = −31.2, IQR = −43.1 to −15.1) within the identified cluster time-window, *Z* = −0.48, *p* = 0.63, *r* = −0.054 (see Figure 4A). HRV was significantly higher at pre-VH compared to post-VH in all cases, whilst RMSSD was significantly increased at pre-VH in comparison to post-VH in the total sample and for the younger group. HR did not differ significantly across conditions in total or within the two subgroups.

**TABLE 1:** Wilcoxon signed-rank comparisons for mECG, HRV, RMSSD and HR within VH conditions.

	VH condition					
	pre-VH	post-VH				
	Mdn (IQR)	Mdn (IQR)	*n*	*Z*	*r*	*p*
**mECG**						
Total	4.53 (−4.50 to 14.7)	2.01 (−6.64 to 11.8)	76	**2.62**	0.21	0.009
Young	4.42 (−1.89 to 14.5)	1.35 (−7.39 to 10.5)	36	**2.55**	0.30	0.011
Elderly	5.82 (−5.65 to 17.2)	2.49 (−6.35 to 12.9)	40	1.18	0.13	0.24
**HRV**						
Total	2.85 (1.73–5.20)	2.46 (1.61–3.76)	76	**4.35**	0.35	0.000
Young	5.02 (3.62–6.50)	3.63 (2.92–5.21)	36	**3.55**	0.42	0.000
Elderly	1.77 (1.31–2.67)	1.65 (1.26–2.02)	40	**2.58**	0.29	0.010
**RMSSD**						
Total	28.2 (14.5–42.8)	24.0 (14.5–39.5)	76	**2.21**	0.18	0.027
Young	33.5 (28.1–50.0)	32.0 (22.0–42.2)	36	**2.37**	0.28	0.018
Elderly	15.4 (11.7–30.0)	15.1 (11.3–32.5)	40	0.78	0.087	0.44
**HR**						
Total	69.6 (60.6–77.2)	70.2 (62.0–78.2)	76	−1.92	−0.16	0.055
Young	72.4 (61.2–78.2)	71.7 (66.0–79.5)	36	−1.48	−0.17	0.14
Elderly	66.6 (59.1–75.6)	66.8 (59.5–75.7)	40	−1.25	−0.14	0.21

Mdn represents the median value, IQR refers to the interquartile range values and *n* indicates the number of participants included in the respective statistical comparison. *Z* represents the *Z*-statistic of the Wilcoxon signed-rank test. The value *r* represents the correlation coefficient and *p* indicates the probability value. Significant results are presented in bold.

### Age group comparisons

#### HEP

In total, cluster-based permutation analyses indicated an effect of age (*p* < 0.001) during pre-VH. This corresponded to a significant positive cluster from 310 to 365 ms, mainly distributed over parietal and left temporal sites (see Supplementary Figure A1). Peak positive HEP difference was observed at 330 ms after R-peak (electrode T3), *t*(74) = 4.04, *p* < 0.001. Mean HEP amplitude averaged across the cluster was significantly higher for young, compared to elderly, participants, *t*(74) = 5.13, *p* < 0.001, *d* = 1.19 (see Supplementary Figure A2). An additional cluster permutation test indicated an effect of sex for post-VH. A significant positive cluster (*p* < 0.001) from 290 to 360 ms was identified, spread over left centroparietal and temporal regions (see Supplementary Figure A3), with peak positive HEP difference obtained at 305 ms after R-peak (electrode T3), *t*(74) = 5.08, *p* < 0.001. Mean HEP amplitude averaged across the cluster was significantly higher for young, compared to elderly, participants, *t*(74) = 5.24, *p* < 0.001, *d* = 1.21 (see Supplementary Figure A4). A significant negative cluster (*p* = 0.013) from 280 to 305 ms was also detected, spread predominantly over right temporal sites and centered over electrode F8 (see Supplementary Figure A3), where peak negative HEP difference was observed 300 ms after R-peak, *t*(74) = −4.00, *p* = 0.013. Mean HEP amplitude averaged across the cluster was significantly higher for older, compared to young, participants, *t*(74) = −4.03, *p* < 0.001, *d* = −0.93 (see Supplementary Figure A4). No interaction effect of age and condition was observed.

#### Control analyses

In general, although there were no significant differences between age groups in mECG within the TOI (see Supplementary Table A2), mECG was significantly lower for young (Mdn = −17.6, IQR = −33.3 to 1.36), compared to elderly (Mdn = 12.8, IQR = −8.49 to 44.5), participants within the pre-VH cluster time-window, *U* = 356, *Z* = −3.78, *p* < 0.001, *r* = −0.43 (see Figure 4B), as well as within the positive post-VH cluster time-window (see Figure 4C), *U* = 303, *Z* = −4.33, *p* < 0.001, *r* = −0.50 (young: Mdn = −30.5, IQR = −44.1 to −7.13; elderly: Mdn = 6.71, IQR = −13.0 to 34.0). Within the negative post-VH cluster time-window, mECG was not significantly different between young (Mdn = 61.0, IQR = 15.8–119) and senior (Mdn = 102, IQR = 56.5–139) participants, *U* = 553, *Z* = −1.73, *p* = 0.083, *r* = −0.20. HRV and RMSSD were significantly higher for young individuals in both VH conditions, while HR did not differ significantly between age groups.

### Sex group comparisons

#### HEP

For the total sample, a cluster permutation test indicated an effect of sex (*p* = 0.021) succeeding VH, corresponding to a significant positive cluster from 560 to 585 ms, mainly distributed over central regions (see Supplementary Figure A5). Maximum positive HEP difference was observed at 570 ms after R-peak (electrode Pz), *t*(74) = 3.56, *p* = 0.021. Mean HEP amplitude averaged across the cluster was significantly higher for males compared to females, *t*(74) = 3.89, *p* < 0.001, *d* = 0.90 (see Supplementary Figure A6). No significant cluster permutation differences were found between sexes within the young and elderly subgroups.

#### Control analyses

Supplementary Table A3 provides an overview of sex-group comparisons for ECG measures. Overall, there were no significant differences in mECG. Moreover, in the total sample, mECG did not differ significantly between males (Mdn = −35.9, IQR = −54.3 to −21.3) and females (Mdn = −30.2, IQR = −50.8 to −16.3) within the post-VH cluster time-window, *U* = 635, *Z* = −0.89, *p* = 0.37, *r* = −0.10 (see Supplementary Figure A7). HRV was generally higher for females during pre-VH. For young participants, that was also the case following VH. No significant RMSSD differences were observed. Lastly, there were no significant HR dissimilarities between sexes for pre-VH for the elderly subgroup, while HR was significantly increased for females within both VH conditions in the total sample.

## Discussion

The present study aimed at investigating HEP responses following a VH intervention in epilepsy patients. Whilst the main analysis over the total sample suggested no difference across pre-VH and post-VH, there was a modulation of HEP as a function of VH condition observed within the elderly subgroup, but in the opposite direction than hypothesized. Specifically, HEPs for elderly participants were higher during pre-VH as compared to post-VH in a time window of 530 to 560 ms after the R-peak, denoting decreased cardiac interoceptive processing following breathing disruptions in senior individuals. This effect was spread predominantly over frontal sites, suggesting variations in visceral, as opposed to somatosensory, afferent representation of cardiac information (Schandry and Montoya, 1996). Importantly, the late timing of detected differences and the lack of discrepancies in ECG amplitude across VH conditions for senior participants, as well as within the time window of the identified cluster, favor the interpretation of acquired variations in HEP amplitude in terms of altered cardiac interoception as opposed to fluctuations in cardiac dynamics (Park and Blanke, 2019) or mechanical cardiorespiratory coupling (Billman, 2011).

With respect to the overall null effect, potential explanations might relate to the confounding influence of various mental (e.g., shifts in attention and/or emotional state) and physiological (e.g., changes in bodily arousal) processes on the HEP (Park and Blanke, 2019; Petzschner et al., 2019; Coll et al., 2021) that could result in fluctuations of the HEP measure at the individual level, thereby contaminating true effects (Verdonk et al., 2021). Indeed, recently, HEP’s reliability over time has been called into question by a study on trait mindfulness and interoceptive sensibility, showing poor HEP reliability across experimental sessions during rest and lack of reliable associations to self-reported body awareness, possibly relating to fluctuations in cardiac arousal at rest (Verdonk et al., 2021). This raises methodological concerns with regards to the validity of the relationship between HEPs and interoception (Coll et al., 2021), especially when it comes to resting-state measurements (Verdonk et al., 2021). Nonetheless, an alternative explanation of the null finding could pertain to variability across subgroups with respect to age-related differences on interoceptive (Murphy et al., 2018; Kamp et al., 2021), as well as autonomic (Voss et al., 2015), processes that, in turn, could have affected statistical power (Kananen et al., 2020). The inclusion of a control group, as well as of increased sample sizes (Goulet and Cousineau, 2019), in future studies could possibly aid in resolving the point of issue.

In light of the foregoing considerations, although speculative, observed reductions in HEP amplitude following VH for elderly participants might reflect the interference of VH-elicited sensory signals from respiratory afferents (Lee and Yu, 2014) with the transmission of cardiovascular ascending information, thereby, resulting in decreased cardiac signaling at the cortical level (MacKinnon et al., 2013). In particular, VH might modify respiratory-related neural input through changes in carbon dioxide (CO_2_) sensing (Guyenet and Bayliss, 2015), as breathing during VH progressively eliminates CO_2_ from the lungs, forcing arterial CO_2_ to drop below normal levels, a phenomenon known as hypocapnia (Godoy et al., 2017). In turn, altered ventilatory signals may interact with cardiac afferents during the integration of interoceptive information (Critchley and Harrison, 2013), thus, modulating HEPs (Baumert et al., 2015). Notably, respiratory interoception might be disrupted for elderly participants, since aging is associated with a progressive decline in lung physiology and function (Sharma and Goodwin, 2006) that could potentially affect CO_2_ sensing (Dempsey and Smith, 2014). Indeed, elderly individuals exhibit altered breathing patterns and arterial CO_2_ tension during exercise hyperpnea, maintaining CO_2_ levels at resting normocapnic range by increasing minute ventilation in order to achieve a CO_2_ exchange ratio comparable to that of younger subjects (Dempsey and Smith, 2014). This suggests alterations in the sensing mechanism of respiratory CO_2_ exchange that could be implicated in homeostatic deficiencies and corresponding CO_2_ afferent signaling (Guyenet and Bayliss, 2015). At the same time, as aging is associated with autonomic imbalances (Voss et al., 2015), compensatory mechanisms responding to pulmonary stressors might be impaired in older individuals (Porta et al., 2014), possibly occasioning breathing instability (Dempsey and Smith, 2014) after VH cessation in elderly individuals. Accordingly, altered CO_2_ sensing could interact with sympathovagal dysautonomia in elderly participants, thereby prolonging respiratory signaling after VH cessation and consequently resulting in diminished cardiac interoceptive processing, possibly also signifying disturbances in overall cardiorespiratory coupling (Druschky et al., 2020).

With regards to control analyses, HRV was significantly lower after VH cessation in both subgroups, in line with HRV-related autonomic imbalances observed post-VH (Neginhal et al., 2017; Sinha et al., 2020). Notably, though, alterations in HEP waveforms seem to generally be independent of changes in HRV (MacKinnon et al., 2013; Luft and Bhattacharya, 2015; Schulz et al., 2015; Marshall et al., 2017; Lutz et al., 2019; Kamp et al., 2021), suggesting that HEPs do not represent variability in cardiac dynamics, but rather interoceptive processes that may be differentially recruited (MacKinnon et al., 2013; Luft and Bhattacharya, 2015) in response to HRV fluctuations (Park and Blanke, 2019). Consistent with this assertion, there were no variations in HEP waveforms within the younger subgroup. In contrast, RMSSD, an index of vagally-mediated alterations in HRV (McCraty and Shaffer, 2015), was significantly lower after VH for young participants, suggesting a post-VH shift toward sympathetic control in young epilepsy adults that is consistent with similar observations in young healthy participants (Sinha et al., 2020), whilst indicating disrupted autonomic cardiac control for elderly individuals (Voss et al., 2015). However, as it has been previously reported that RMSSD is not correlated with HEP amplitude during rest, emotional stimulation, or breathing manipulations (MacKinnon et al., 2013), it is unlikely that RMSSD differences alone drive the incongruencies in HEP results acquired for the young and elderly subgroups.

Assuredly, concerning prospective caveats, the potential influence of changes in cardiac dynamics, even to a partial extent, on the acquired HEP modulations as a function of VH cannot be readily excluded (Coll et al., 2021), given also the obtained variation in HRV across VH conditions within the elderly subgroup. Importantly, though, the generation of HRV is a highly complicated process involving the regulation of vagal afferents by the intrinsic nervous system of the heart, as well as their integration with autonomic efferent signals within innately produced cardiac activity (McCraty and Shaffer, 2015). Therefore, variations in HRV might indicate disruptions in overall neurocardiac function, involving both altered heart-dependent signaling (MacKinnon et al., 2013) and sympathovagal fluctuations (Neginhal et al., 2017; Sinha et al., 2020). Consistent with this speculation, behavioral studies (Knapp-Kline and Kline, 2005; Owens et al., 2018; Leganes-Fonteneau et al., 2021) linking various HRV measures to heartbeat detection tasks support the interrelatedness of HRV with the perception of interoceptive cardiac information, which is, in turn, connected to HEP responses (Park and Blanke, 2019), although the precise role of HRV in afferent cardiac communication remains obscure (Coll et al., 2021). Still, there is evidence that HRV may be implicated in regulating the strength of the connection between the heart and the brain (MacKinnon et al., 2013), perhaps by modulating interferences in ascending vagal pathways from heart-related autonomic efferent information through baroreflex functioning (Rahman et al., 2011; Leganes-Fonteneau et al., 2021) to ensure the maintenance of homeostasis (Critchley and Harrison, 2013), thus affecting cardiac afferent traffic (McCraty and Shaffer, 2015). Of late, however, it has been also proposed that interoceptive afferents might control the mediation of homeostasis by the autonomic system and hence moderate HRV parameters (Owens et al., 2018), which could be associated with the capacity to engage autonomic reflexes in response to stressors (Owens et al., 2018; Park and Blanke, 2019). Crucially, HRV assessment in this study was based on a geometric method (Vollmer, 2015) that is more robust against breathing-induced HR changes compared to spectral and time-domain metrics (Billman, 2011; Shaffer and Ginsberg, 2017), including RMSSD (Druschky et al., 2020; Sinha et al., 2020), suggesting that acquired HRV differences indeed represent changes in neurocardiac dynamics rather than fluctuations in ventilation parameters.

With respect to age-group comparisons, most results were in the predicted direction, with young participants displaying higher HEPs than older adults at early time points both preceding (310–365 ms after R-peak) and succeeding (290–360 ms after R-peak) VH performance. The detected effects were distributed over left parietal and temporal regions, possibly indicating decreased somatosensory afferent processing in elderly subjects (Malandraki et al., 2011). Upon initial inspection, these findings are inconsistent with those obtained in one recent study (Kamp et al., 2021), published after the preregistration of the current research, where older adults exhibited increased HEPs at rest, compared to young adults, in a community sample. This age-related effect was observed in a late temporal window (455–595 ms after R-peak) and over frontal areas, denoting increased visceral, as opposed to somatosensory, afferent processing (Schandry and Montoya, 1996) and, in that sense, matching the acquired HEP effect as a function of VH condition within the elderly subgroup. Although this discordance in findings may relate to the use of dissimilar populations and/or HEP analysis paradigms, it is important to note that there were confounding differences in ECG amplitude for observed HEP differences between age groups in the present study, possibly relating to confounding CFA influences, so it is unclear whether acquired HEP differences between age groups represent age dissimilarities in cardiac parameters *per se*, early age-related variations in the neural processing of cardiac signals, or a combination of both (Kamp et al., 2021). In any event, obtained findings match the posterior HEP waveform increases in earlier time intervals (180–320 ms after R-peak) observed for young participants at rest by Kamp et al. (2021), though early age differences were no longer significant after controlling for CFA in their study, suggesting a major CFA influence for detected HEP modulations as a function of age group in the current case. Admittedly, the only acquired age differences that appear least confounded by the CFA, as evident by the lack of variation in ECG amplitude between age groups within the cluster time-window, concern the augmentation in HEP waveforms at 280–305 ms after R-peak for older individuals succeeding VH termination, with this effect spread over right temporal sites and a single right frontal electrode. This is in agreement with similar CFA-uncorrected findings for elderly subjects in early temporal intervals (Kamp et al., 2021), but again not entirely dissociable from CFA-related noise. Still, despite the negative correlation of HR with HEP amplitude at rest observed in older, but not younger, age (Kamp et al., 2021), there were no HR differences between age groups at baseline or after VH cessation, signifying the existence of additional sources of variability between findings in the aforementioned and present study.

Concerning sex, findings were in the opposite direction than anticipated. In total, males exhibited higher HEPs at 560–585 ms after R-peak compared to females following VH, suggesting augmented cardiac interoception for male participants after breathing perturbations. This contradictory finding may relate to the investigation of a clinical group, as opposed to a healthy sample (MacKinnon et al., 2013; Grabauskaitė et al., 2017). Alternatively, it may reflect the confounding influence of age-dependent differences in female sex hormones that are associated with altered baroreceptor sensitivity (Fu and Ogoh, 2019), which could also explain the partial overlap in observed variability between sex groups. Indeed, the time interval and location of the effect conform to the central representation of baroreceptor-mediated cardiac afferents (Gray et al., 2007). In any case, however, the late timing of detected differences and lack of variation in ECG amplitude between sex groups succeeding VH, as well as within the time window of the identified cluster, suggest that the identified alterations in HEP amplitude reflect genuine neural activity rather than mere cardiac artifacts. At the same time, acquired HR differences are consistent with higher HR reports in healthy women compared to age-matched healthy men (Moodithaya and Avadhany, 2012), yet do not mirror post-VH variations in HEP amplitude, so it is unlikely that the obtained effect represents dissimilarities between sexes in cardiac fluctuations. So, overall, acquired HEP modulations seem to verily index variations in cardiac interoception, independent of whether they actually reflect age-or sex-related differences, although additional research, involving healthy control groups and monitoring of hormonal influences, is necessary in order to unravel the actual mechanism underlying the obtained effects.

## Limitations

Since this preliminary study was meant to be a retrospective exploration of an epileptic patient group, aiming to establish the core rationale for further investigation of interoceptive neurobiological markers in clinical populations, the inclusion of a healthy population group in future studies would be mandatory to control for extraneous factors (Hunter et al., 2014). Besides not including a control group, our study did not incorporate an explicit measure of respiratory interoception, thereby focusing on the effects of altered respiratory patterns, rather than breathing-related interoceptive signaling *per se* (von Leupoldt et al., 2010a), on cardiac neural processing. Such a metric would have been conducive in further exploring the relatedness of breathing to cardiac interoception. Notably, a perceptual threshold breathing and metacognition task, termed the Filter Detection Task, has been developed recently for the quantification of breathing-related interoceptive dimensions that can be easily integrated in clinical settings due to its simple setup (Harrison et al., 2021) and, thus, may constitute an important asset for future studies. Interestingly, effects of breathing on HEP amplitude appear to be modulated by respiratory phase, at least in spontaneous breathing conditions, with increased HEP waveforms observed during exhalation in healthy adults at rest, indicating an optimization of interoceptive cortical processing of heart-related signaling across the ventilatory cycle (Zaccaro et al., 2022). On the other hand, a study showing decreased HEPs during expiration in children with sleep-disordered breathing (Baumert et al., 2015) suggests this optimizing mechanism might be impaired in clinical conditions accompanied by alterations in cardiorespiratory interactions. In this regard, it would be interesting to investigated whether HEP responses are respiratory phase-dependent in altered breathing conditions in healthy, as well as in clinical populations.

Finally, further restrictions could relate to the confounding nature of VH due to its cerebro-vascular effects on brain physiology and metabolism (Gouvea Bogossian et al., 2021). Indeed, HEP amplitude during resting-state in magnetoencephalography measurements co-varies with stroke volume differences over occipital and bilateral occipito-temporal areas in the time-window of 500–700 ms post R-peak (Buot et al., 2021). This effect disappeared with an ICA correction procedure of cardiac components (Oostenveld et al., 2011), although these results may vary depending on the statistical computation as well as HEP recording method applied (Buot et al., 2021). It is, moreover, unclear, whether the relationship between ICA-uncorrected HEPs and stroke volume is indeed mediated by the cardiac artifact or if it reflects genuine neural activity dependent on stroke volume due to transient arterial baroreceptor activity masked by the ICA correction (Buot et al., 2021). At the same time, changes in respiration rhythm affect cortical hemodynamics and thus may act as a physiological confounding factor in heart-related interoceptive awareness (Candia-Rivera et al., 2022), at least when applying the heartbeat-counting task (Desmedt et al., 2018). On the other hand, as the heartbeat-counting task may be contaminated to a large extent by non-interoceptive processes (Desmedt et al., 2018), it remains unclear whether fluctuations in breathing rate primarily affect cardiac interoception and, if so, to what extent, warranting thereby further exploration, whilst carefully controlling for differences in physiological parameters between conditions. In this regard, additional study limitations comprise the lack of pulmonary measures, such as breathing frequency, as well as the absence of capnographic evaluation, since such assessments are not incorporated in the usual clinical EEG (Sinha et al., 2016). It is unclear how respiratory rate influences RMSSD (Shaffer and Ginsberg, 2017), while literature regarding ventilatory effects on the HRV metric implemented in this study is, to the knowledge of the authors, lacking. So, the inclusion of such an estimate would be meaningful for controlling potential pulmonary confounds, as well as quantitatively appraising VH execution.

Nonetheless, breathing frequency alone does not guarantee that VH is effectively performed, as hyperventilation due to increases in minute ventilation can also ensue from heightened air tidal volume while pulmonary rate remains unaffected (Ritz et al., 2009). In this regard, CO_2_ metrics would have been beneficial in determining the VH level achieved (Coppola et al., 2010), as well as in linking CO_2_ decreases to HEP amplitude. Still, the omission of capnographic assessment should not adversely affect acquired HEP results, as changes in VH-induced EEG activity do not seem to differ substantially between CO_2_-controlled and routine VH (Zwiener et al., 1998). Furthermore, routine VH during clinical EEG examinations appears to evoke seizures or exacerbation of interictal discharge rather infrequently (Holmes et al., 2004; Kane et al., 2014; Alghamdi et al., 2021), whilst the mechanism underlying this phenomenon remains unclear (Salvati and Beenhakker, 2019). There is, in one respect, evidence that VH-elicited changes in EEG recordings involve patient-specific sensitivities to CO_2_ sensing (Salvati et al., 2022; Rana et al., 2023), although the absolute values of partial arterial CO_2_ seem to be less significant than the acute changes in blood CO_2_ when it comes to the provocation of spike-wave activity (Son et al., 2012). Thus, it remains unknown whether concrete arterial CO_2_ levels are involved in VH-induced epileptiform discharges. In any case, the association between VH-specific EEG observations and CO_2_ changes is still debated (Rana et al., 2023) and may involve additional factors, including sympathovagal imbalances (Lotufo et al., 2012; Assenza et al., 2015; Bermeo-Ovalle et al., 2015) and cardiorespiratory dysfunction (Akyüz et al., 2021).

## Outlook

Despite the abovementioned limitations in study design, our findings suggest a modulatory effect of altered breathing on the neural processing of heart-related interoceptive signaling in epilepsy patients. HEPs could, thus, provide a valuable marker of aberrant neurocardiac interactions in epilepsy that may be of particular relevance for clinical symptoms, such as functional seizures (Koreki et al., 2020) or sudden unexpected death in epilepsy (Moseley and Britton, 2014), possibly relating to systemic dysfunction within the cardiovagal circuitry (Druschky et al., 2020) and overall sympathetic overactivity (Myers et al., 2018). The transdiagnostic role of HEPs in other neuropsychiatric conditions and how that diverges from healthy neurobiological processes remains to be explored. In any case, due to VH’s physiologically activating nature it is difficult to dissociate neurocardiac interactions from sympathetically-driven effects, so further research is necessary before drawing any definitive conclusions. In that respect, it would be helpful to investigate alternative VH protocols, such as prolonged VH interventions (Craciun et al., 2015) and post-VH measurements (Son et al., 2012; Neginhal et al., 2017), and how these relate to VH-induced HEP alterations. The exploration of cardiorespiratory dysautonomia through stimulation of non-chemosensory peripheral channels (Hassanpour et al., 2016, 2018) and its potential implications in HEP modulations by breathing could (Dick et al., 2014b) also possibly help in the disentanglement of CO_2_-mediated and autonomically-prompted effects on cardiac interoception. Additionally, it would be important to examine other forms of aberrant respiration and their relationship to cardiac interoception, like dyspnea, a CO_2_-related interoceptive breathing sensation linked to several mental irruptions (Allard et al., 2017; Vinckier et al., 2018). Investigation of the effects of cardiovascular input on respiratory interoception would also be beneficial, as there is accumulating evidence that baroreceptor activation can modulate breathing (Barnett et al., 2021).

Finally, epilepsy subcategories generally differ in terms of viscero-vegetative and somatosensory symptomatology (Aljafen, 2020), while they present variations in autonomic regulation (Myers et al., 2018) and VH-triggered epileptiform discharges (Guaranha et al., 2005; Abubakr et al., 2010) as well. In that sense, different epilepsy forms could also vary with regards to interoceptive irregularities. Psychogenic seizures, for instance, are associated with distinct disruptions in interoceptive processing that predict both dissociative symptoms and the occurrence of functional seizures (Koreki et al., 2020). In that respect, interoceptive neural markers, such as the HEP, could potentially constitute valuable diagnostic tools in the identification of interoceptive abnormalities that may be related to various epilepsy subtypes (Petzschner et al., 2019). Of note, insular epilepsy (Jobst et al., 2019), an underrecognized epilepsy form and great mimicker of psychogenic non-epileptic seizures and other epilepsies with temporal semiology, could be of particular interest to future studies on cardiorespiratory interoception due to the implications of the insular cortex in the representation of concurrent changes in cardiorespiratory states (Hassanpour et al., 2016, 2018) and overall interoceptive processing (Azzalini et al., 2019), as well as its potential involvement in seizure-related cardiopulmonary dysfunction and associated patho phenomenology of sudden unexpected death in epilepsy (Jobst et al., 2019).

Concluding, breathing constitutes a vital interoceptive dimension, which possesses a fundamental role in driving neuronal activity (Heck et al., 2017; Herrero et al., 2018), possibly also relating to the modulation of cortical activity by cardiac afferent signals (McCraty and Shaffer, 2015). The present study contributes to current knowledge surrounding the interplay of breathing and cardiac interoception by providing preliminary evidence of ventilatory-related HEP modulations in elderly epilepsy patients. This could have considerable implications for HEP’s clinical significance as a prospective biomarker in the assessment of aberrant interoceptive signaling (Khalsa and Lapidus, 2016), establishing the rationale for extending this line of research to large-scale studies in various neuropsychiatric populations. Available databases involving patient records of routine VH assessment may constitute a valuable asset in this regard, whilst the application of software, such as Automagic (Pedroni et al., 2019), would allow the automated processing of large EEG data. Notably, as ventilation is situated at the crossroads of exteroception and interoception (Davenport and Vovk, 2009), a potential translatability of HEP waveforms to respiratory signaling could assist in elucidating the neural underpinnings of their intricate relationship (Marshall et al., 2017; Salvato et al., 2020).

## Data availability statement

The datasets presented in this article are not readily available because according to data protection guidelines patient datasets are not publicly available. Further inquiries can be directed to the corresponding author/s.

## Ethics statement

The studies involving humans were approved by Cantonal Ethics Committee Zurich (approval number: 2019-00890). The studies were conducted in accordance with the local legislation and institutional requirements. The participants provided their written informed consent to participate in this study.

## Author contributions

NS: Data curation, Formal analysis, Investigation, Methodology, Software, Writing – original draft, Writing – review & editing. MW: Conceptualization, Funding acquisition, Methodology, Resources, Software, Supervision, Validation, Writing – review & editing. LI: Conceptualization, Methodology, Project administration, Resources, Supervision, Writing – review & editing. BL: Conceptualization, Funding acquisition, Methodology, Resources, Supervision, Writing – review & editing.
