# Supplementary material for: Heartbeat-evoked potentials following voluntary hyperventilation in epilepsy patients: respiratory influences on cardiac interoception
Source: Front Neurosci. 2024 Jul 5;18:1391437. doi: 10.3389/fnins.2024.1391437 (PMC11259972; doi:10.3389/fnins.2024.1391437)
Supplement: Supplementary file 1 [file Data_Sheet_1.pdf]

Heartbeat-Evoked Potentials Following Voluntary Hyperventilation in Epilepsy Patients: Respiratory Influences on Cardiac Interoception

Niovi A. Stoupi[a1*], Marieke L. Weijs[a2], Lukas Imbach[b,c,d], Bigna Lenggenhager[a3]

[a]Department of Psychology, University of Zurich, Zurich, Switzerland

[b]Department of Neurology, University Hospital of Zurich, Zurich, Switzerland

[c] Swiss Epilepsy Center, Klinik Lengg, Zurich, Switzerland

[d] Zurich Neuroscience Center, University of Zurich and ETH Zurich, Zurich, Switzerland

[1] niovi-alexandra.stoupi@clienia.ch

[2] marieke.weijs@uzh.ch

[3] bigna.lenggenhager@psychologie.uzh.ch

***** Corresponding Author

Supplementary Data

Table A1

Within- and Between-Groups Comparisons of Included Epochs

Group	Repeated Measures						
Group	*Mdn (IQR)*	*Mdn (IQR)*	*n*	*Z*	*r*		
	VH Condition						
	pre-VH	post-VH					
Young	180 (161 – 206)	192 (170 – 199)	36	-1.33	-.16		
Elderly	189 (175 – 214)	193 (175 – 217)	40	-1.70	-.19		
Total	189 (175 – 212)	194 (175 – 213)	76	-1.76	-.14		
	Between-Groups						
	Mdn (IQR)	*n*	*Mdn (IQR)*	*n*	*U*	*Z*	*r*
	Sex						
	Male		Female				
Young							
pre-VH	179 (164 – 204)	19	193 (154 – 207)	17	161	-0.016	-.003
post-VH	190 (171 – 200)	19	193 (165 – 199)	17	161	-0.016	-.003
Elderly							
pre-VH	191 (174 – 214)	20	187 (175 – 213)	20	202	0.041	.006
post-VH	189 (175 – 213)	20	200 (185 – 217)	20	173	-0.73	-.12
Total							
pre-VH	185 (174 – 212)	39	193 (175 – 211)	37	735	0.13	.015
post-VH	193 (175 – 204)	39	200 (178 – 216)	37	644	-0.80	-.092
	Age						
	Young		Elderly				
Total							
pre-VH	189 (173 – 211)	36	189 (175 – 214)	40	664	-0.58	-.067
post-VH	195 (176 – 205)	36	193 (175 – 217)	40	643	-0.80	-.092

Note. Repeated measures refer to Wilcoxon signed-rank tests across VH conditions. Between-groups refer to Wilcoxon rank-sum tests for sex and age groups. *Mdn* represents the median value, *IQR* refers to the interquartile range values and *n* indicates the number of participants. *U* represents the Mann-Whitney U-test statistic for Wilcoxon rank-sum tests. *Z* for repeated measures and for between-groups represents the Z-statistic for Wilcoxon signed-rank and rank-sum comparisons, respectively. The value *r* represents the correlation coefficient for the Wilcoxon signed-rank and rank-sum analyses.

Table A2

Wilcoxon Rank-Sum Comparisons for ECG Measures Between Age Groups

		Young		Elderly				
		Mdn (IQR)	*n*	*Mdn (IQR)*	*n*	*U*	*Z*	*r*
mECG	pre-VH	4.42 (-1.89 – 14.5)	36	5.82 (-5.65 – 17.2)	40	732	0.12	.014
	post-VH	1.35 (-7.39 – 10.5)	36	2.49 (-6.35 – 12.9)	40	678	-0.43	-.050
HRV	pre-VH	5.02 (3.62 – 6.50)	36	1.77 (1.31 – 2.67)	40	1231	**5.31****	.61
	post-VH	3.63 (2.92 – 5.21)	36	1.65 (1.26 – 2.02)	40	1296	**5.99****	.69
RMSSD	pre-VH	33.5 (28.1 – 50.0)	36	15.4 (11.7 – 30.0)	40	1100	**3.95****	.45
	post-VH	32.0 (22.0 – 42.2)	36	15.1 (11.3 – 32.5)	40	1072	**3.66****	.42
HR	pre-VH	72.4 (61.2 – 78.2)	36	66.6 (59.1 – 76.0)	40	849	1.34	.15
	post-VH	71.7 (66.0 – 79.5)	36	66.8 (59.5 – 75.7)	40	860	1.45	.17

Note. Mdn represents the median value, *IQR* refers to the interquartile range values and n indicates the number of participants included in the respective statistical comparison. *U* represents the Mann-Whitney U-test statistic and *Z* represents the Z-statistic of the Wilcoxon rank-sum test. The value *r* represents the correlation coefficient. * indicates $p < .05$ and ** indicates $p < .001$.

Table A3

Wilcoxon Rank-Sum Comparisons for ECG Measures Between Sex Groups

			Males		Females				
Measure			*Mdn (IQR)*	*n*	*Mdn (IQR)*	*n*	*U*	*Z*	*r*
mECG	pre-VH	Young	6.88 (0.44 – 17.2)	19	2.37 (-2.95 – 10.2)	17	201	1.24	.21
		Elderly	9.34 (-5.47 – 21.1)	20	-0.66 (-5.65 – 11.3)	20	248	1.29	.20
		Total	9.24 (-3.36 – 19.3)	39	0.44 (-4.77 – 10.5)	37	900	1.85	.21
	post-VH	Young	4.83 (-5.91 – 12.5)	19	0.89 (-7.27 – 6.82)	17	187	0.79	.13
		Elderly	10.5 (-5.98 – 15.7)	20	-0.83 (-6.35 – 8.16)	20	256	1.50	.24
		Total	7.51 (-6.32 – 15.0)	39	0.035 (-6.75 – 6.82)	37	893	1.78	.20
HRV	pre-VH	Young	3.73 (2.37 – 6.48)	19	5.20 (4.96 – 6.44)	17	86	**-2.38***	-.40
		Elderly	1.60 (1.19 – 2.02)	20	2.38 (1.46 – 3.64)	20	124	**-2.04***	-.32
		Total	2.20 (1.57 – 3.79)	39	4.58 (2.14 – 5.64)	37	498	**-2.32***	-.27
	post-VH	Young	3.10 (2.54 – 4.31)	19	4.38 (3.49 – 5.55)	17	96	**-2.06***	-.34
		Elderly	1.56 (1.26 – 1.80)	20	1.72 (1.33 – 2.46)	20	166	-0.91	-.14
		Total	2.16 (1.55 – 3.30)	39	2.87 (1.71 – 4.87)	37	601	-1.25	-.14
RMSSD	pre-VH	Young	29.5 (21.0 – 55.6)	19	38.6 (31.7 – 45.3)	17	119	-1.33	-.22
		Elderly	14.5 (11.7 – 29.3)	20	17.1 (12.0 – 30.8)	20	186	-0.37	-.058
		Total	26.0 (14.3 – 42.1)	39	29.3 (15.5 – 43.7)	37	637	-0.87	-.10
	post-VH	Young	28.7 (18.6 – 41.9)	19	32.3 (26.1 – 45.5)	17	133	-0.89	-.15
		Elderly	16.6 (10.9 – 33.4)	20	14.5 (12.0 – 30.1)	20	195	-0.12	-.019
		Total	20.3 (15.4 – 41.3)	39	25.4 (13.9 – 36.8)	37	688	-0.34	-.039
HR	pre-VH	Young	64.8 (60.5 – 73.3)	19	75.2 (71.0 – 80.2)	17	91	**-2.22***	-.37
		Elderly	61.9 (58.2 – 73.3)	20	70.1 (60.4 – 78.8)	20	142	-1.56	-.25
		Total	62.5 (59.0 – 73.3)	39	73.2 (63.3 – 80.2)	37	477	**-2.54***	-.29
	post-VH	Young	70.1 (63.5 – 77.7)	19	74.6 (69.6 – 80.8)	17	115	-1.46	-.24
		Elderly	63.8 (58.7 – 72.8)	20	72.8 (62.2 – 78.3)	20	142	-1.56	-.25
		Total	66.8 (59.1 – 76.2)	39	74.0 (64.6 – 80.2)	37	527	**-2.02***	-.23

Note. *Mdn* represents the median value, *IQR* refers to the interquartile range values and *n* indicates the number of participants. *U* represents the Mann-Whitney U-test statistic. *Z* represents the Z-statistic of the Wilcoxon rank-sum test. The value *r* represents the correlation coefficient. * indicates $p < .05$.

Figure A1

HEP Topography for Age Groups during Pre-VH

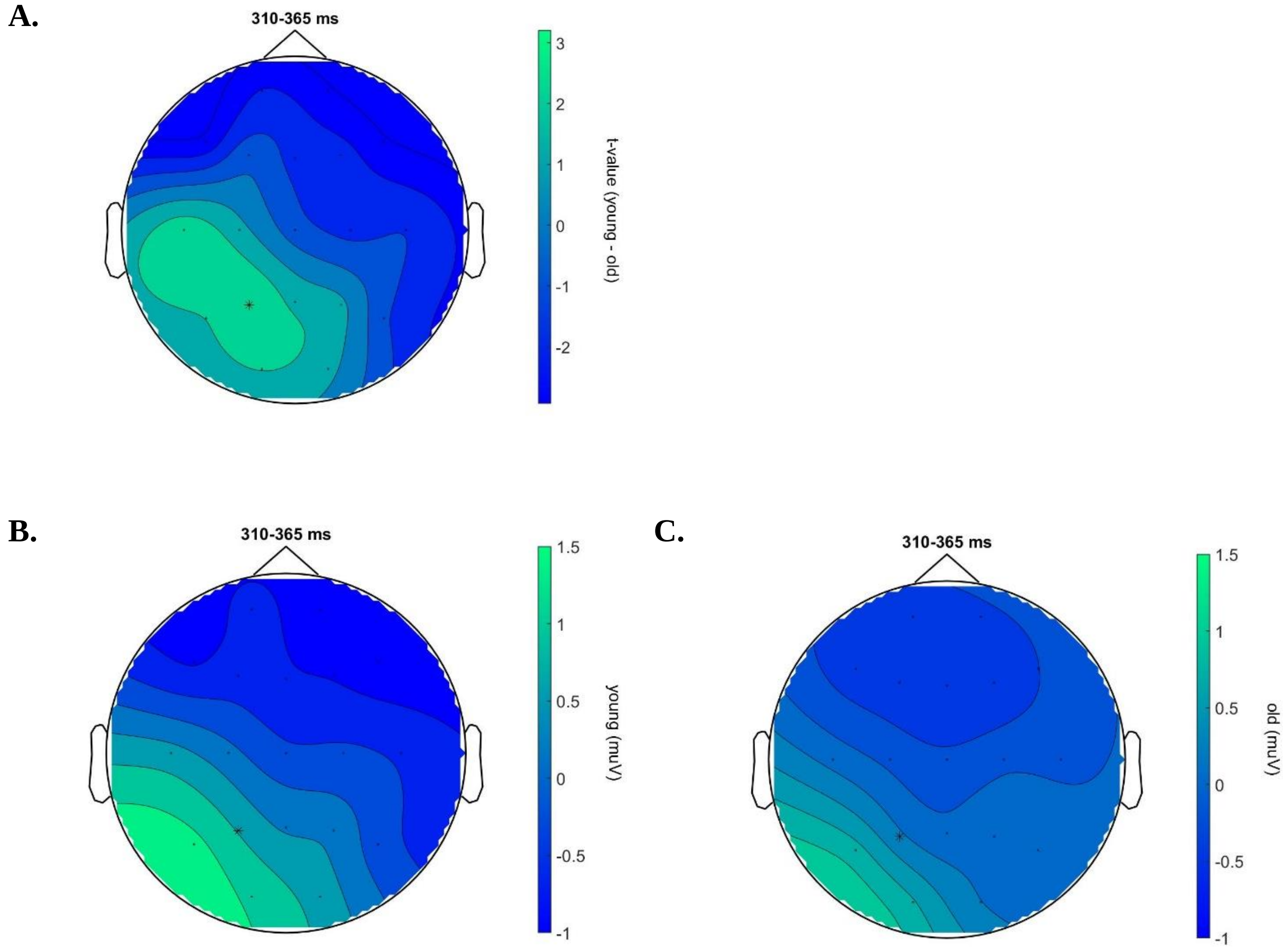

Note. Neural responses to heartbeats between age groups during pre-VH. A) Topography of the identified positive cluster ($p < .001$) depicting the t-values for the age-group comparison at the time window of significant activation (310 – 365 ms). HEP differences at electrode P3, indicated by an asterisk, remained significant throughout the entire cluster time-window. ($p < .001$ at all highlighted electrode sites). B) HEP topography for young participants ($N = 36$) showing mean HEP amplitude, averaged across the TOI, during the cluster time window with electrode P3 highlighted. C) HEP topography for elderly participants ($N = 40$) showing mean HEP amplitude, averaged across the TOI, during the cluster time window with electrode P3 highlighted.

Figure A2

ERP Waveforms for Age Groups in Total during Pre-VH

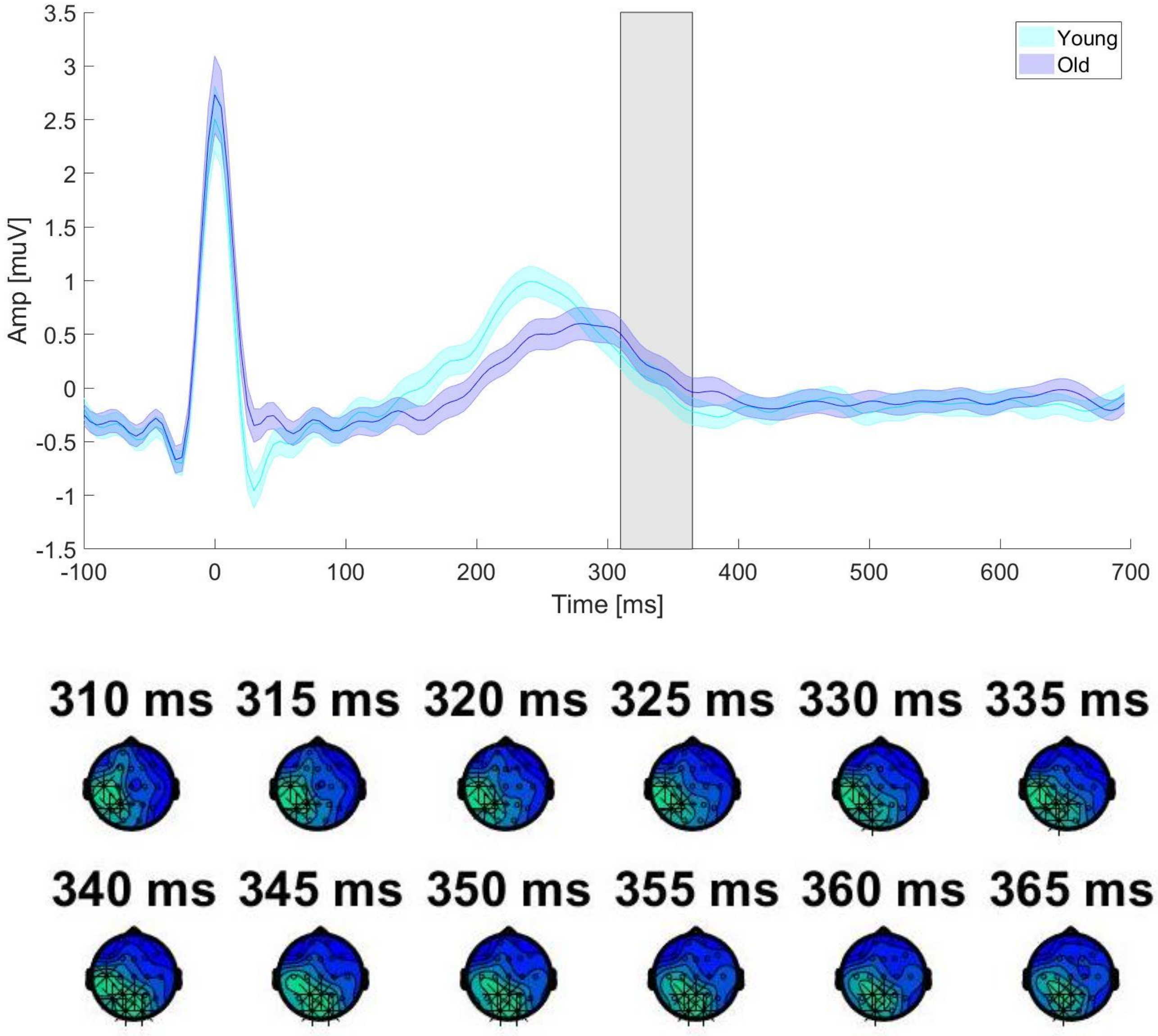

Note. ERP waveforms showing HEP amplitude and standard error of the mean, averaged across all cluster electrodes (P3, O1, O2, T3, T5, Pz, T9) for young (N = 36) and elderly (N = 40) subjects. The gray shaded area represents the time period (310 – 365 ms) in which a significant positive cluster was identified (see Figure A1). The topography below shows the electrodes contributing to the cluster at different time points ($p < .001$ at all highlighted electrode sites) during the time window of significant activation (310 – 365 ms).

Figure A3

HEP Topography for Age Groups during Post-VH

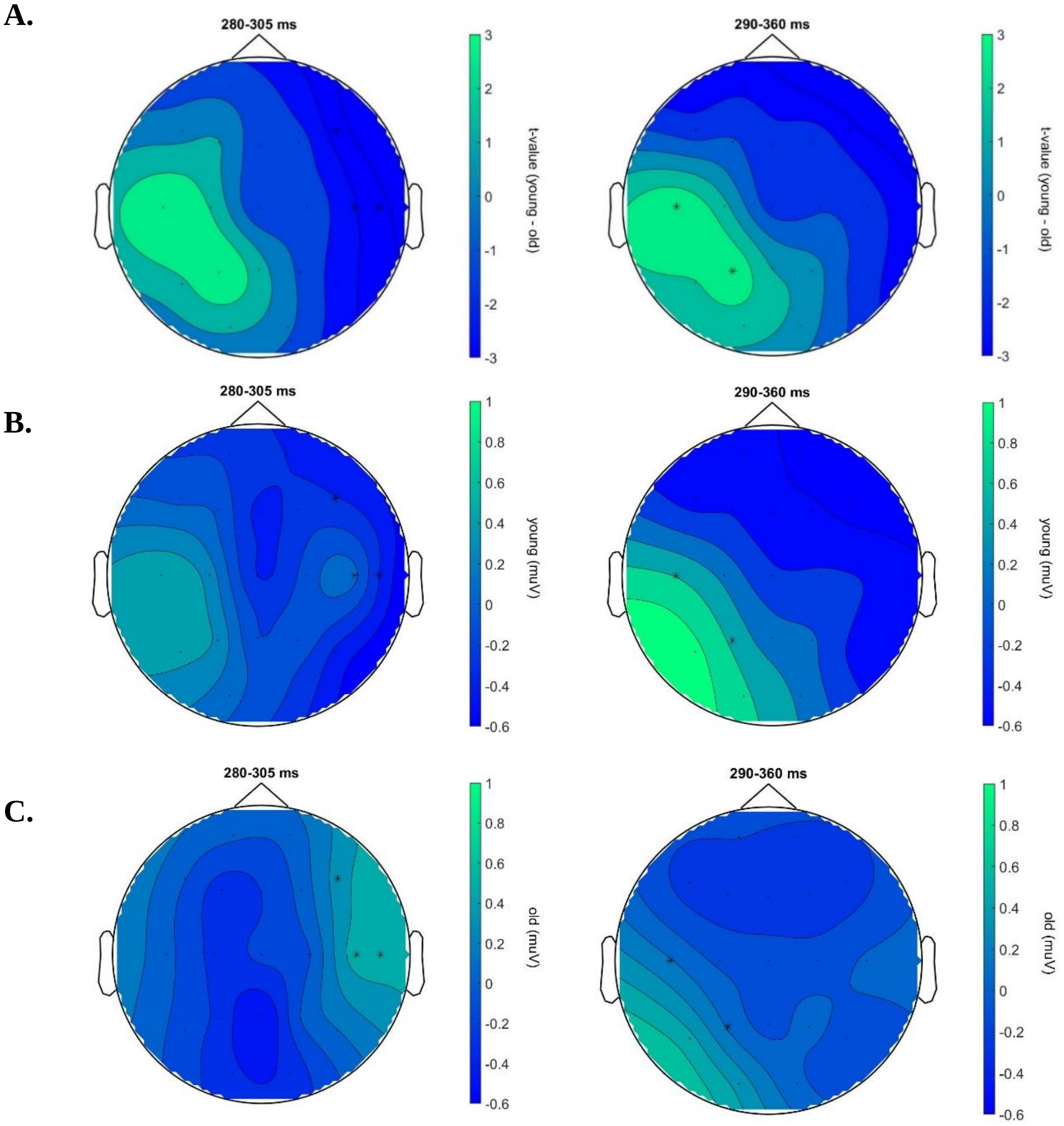

Note. Neural responses to heartbeats between age groups during post-VH. A) Topography of the identified negative (left) and positive (right) clusters depicting the *t*-values for the age-group comparison during the respective time window of significant activation. HEP differences at electrodes F8, T4 and T10 for the negative cluster ($p < .01$ at all highlighted electrode sites) and electrodes C3 and P3 for the positive cluster ($p < .001$ at all highlighted electrode sites), indicated by an asterisk, remained significant throughout the entire cluster time-window. B) HEP topography for young participants ($N = 36$) showing mean HEP amplitude, averaged across the TOI, during the negative (left) and positive (right) cluster time window with respective cluster electrodes highlighted. C) HEP topography for elderly participants ($N = 40$) showing mean HEP amplitude, averaged across the TOI, during the negative (left) and positive (right) cluster time window with respective cluster electrodes highlighted.

Figure A4

ERP Waveforms for Age Groups during Post-VH

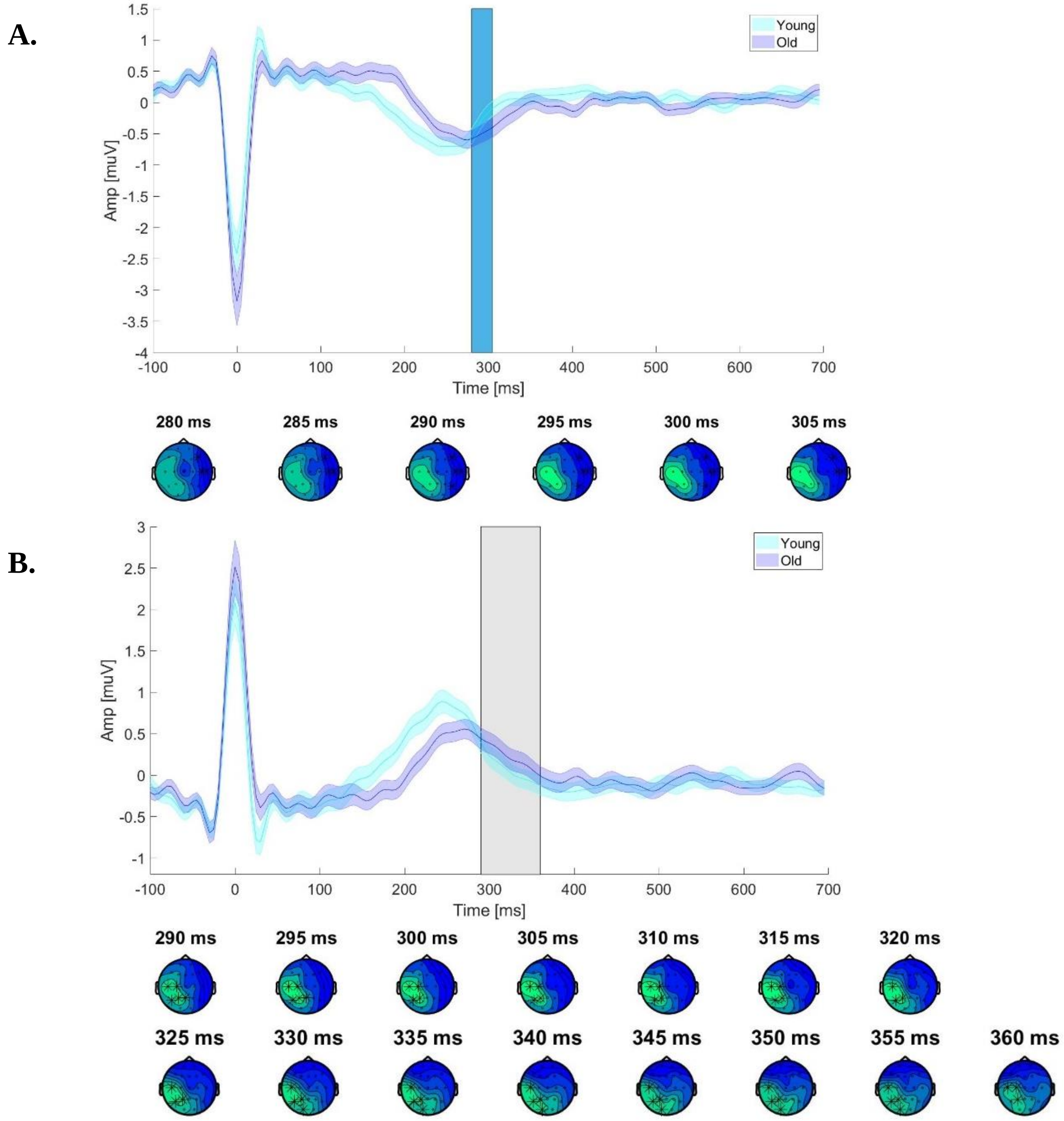

Note. A) ERP waveforms showing HEP amplitude and standard error of the mean averaged across all negative (F8, T4, T6, T10) cluster electrodes for young ($N = 36$) and elderly ($N = 40$) participants. The blue shaded area represents the time period (280 – 305 ms) in which a significant difference was obtained (see Figure A3). The topography below shows the electrodes contributing to the cluster at different time points ($p < .01$ at all highlighted electrode sites) during the time window of significant activation. B) ERP waveforms showing HEP amplitude and standard error of the mean averaged across all positive (C3, P3, O1, T3, T5, Pz, T9) cluster electrodes for young ($N = 36$) and elderly ($N = 40$) participants. The gray shaded area represents the time period (290 – 360 ms) in which a significant difference was obtained (see Figure A3). The topography below shows the electrodes contributing to the cluster at different time points ($p < .001$ at all highlighted electrode sites) during the time window of significant activation.

Figure A5

HEP Topography for Sex Groups in Total during Post-VH

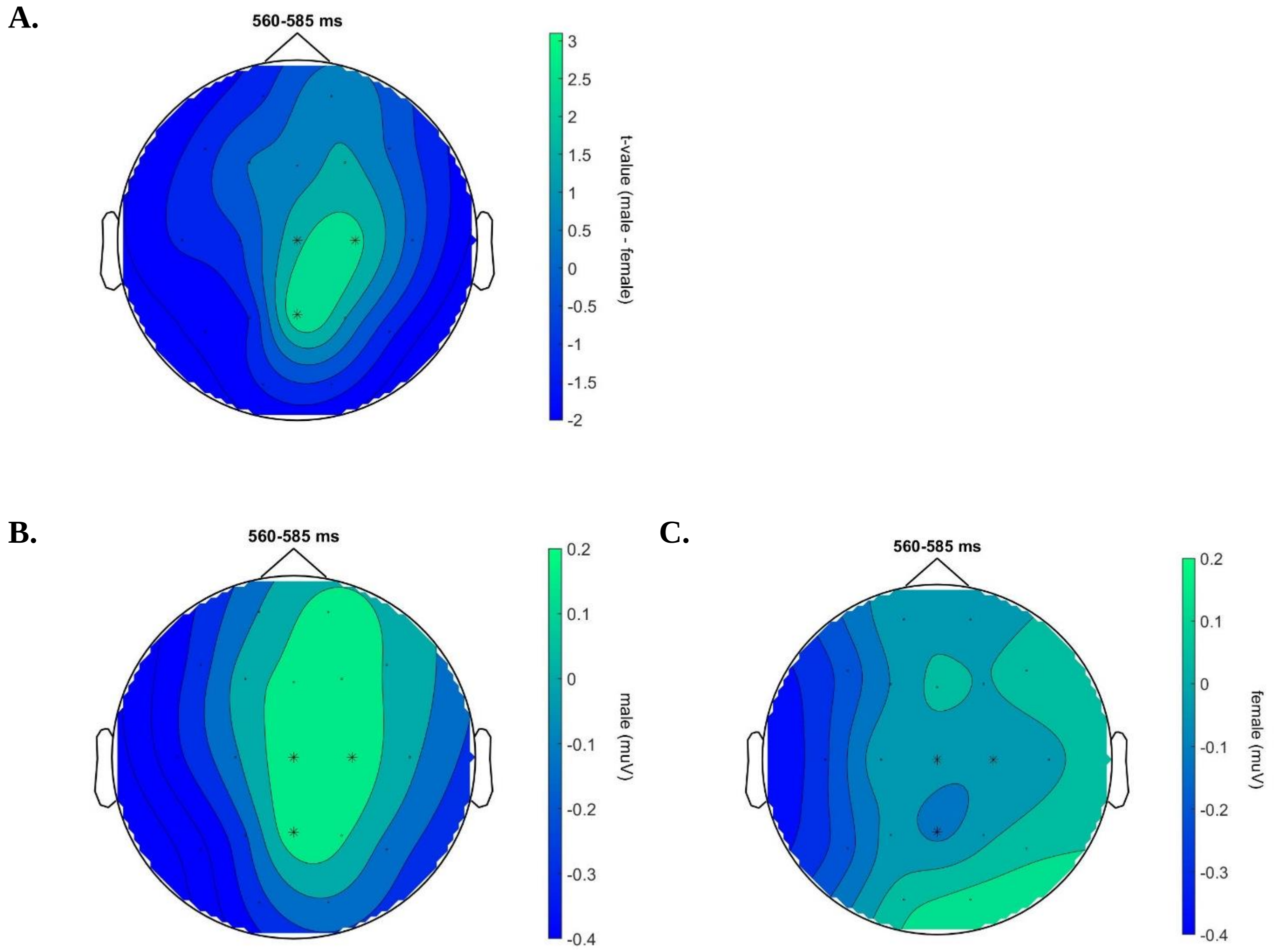

Note. Neural responses to heartbeats between sex groups during post-VH. A) Topography of the identified positive cluster ($p = .021$) depicting the *t*-values for the sex-group comparison during the time window of significant activation (560 – 585 ms). HEP differences at electrodes C4, Cz and Pz, indicated by an asterisk, remained significant throughout the entire cluster time-window. ($p < .05$ at all highlighted electrode sites). B) HEP topography for males ($N = 39$) showing mean HEP amplitude, averaged across the TOI, during the cluster time window with electrodes C4, Cz and Pz highlighted. C) HEP topography for females ($N = 37$) showing mean HEP amplitude, averaged across the TOI, during the cluster time window with electrodes C4, Cz and Pz highlighted.

Figure A6

ERP Waveforms for Sex Groups in Total during Post-VH

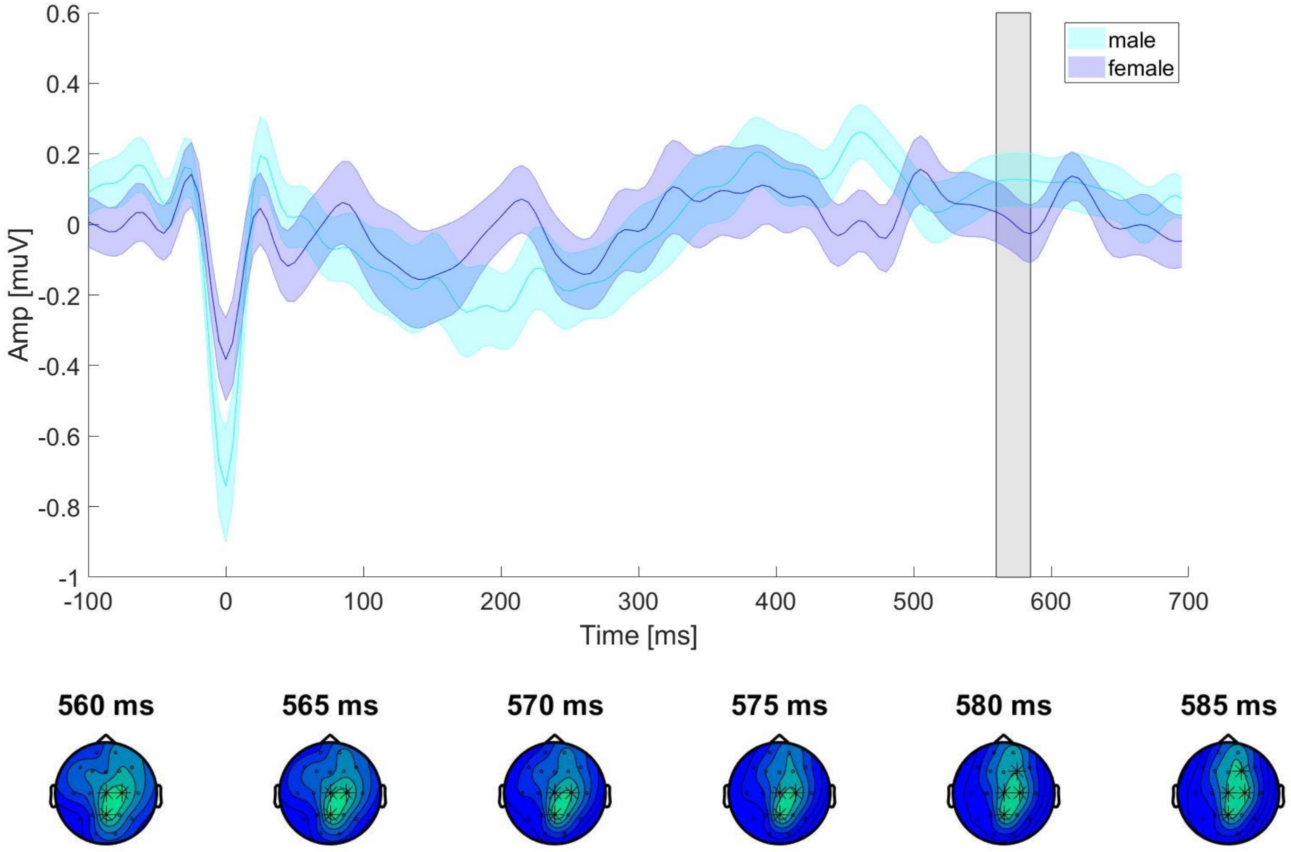

Note. ERP waveforms showing HEP amplitude and standard error of the mean, averaged across all cluster electrodes (F4, C4, Cz, Pz) for male ($N = 39$) and female ($N = 37$) participants. The gray shaded area represents the time period (560 – 585 ms) in which a significant positive electrode cluster was identified (see Figure A5). The topography below shows the electrodes contributing to the cluster at different time points ($p < .05$ at all highlighted electrode sites) during the time window of significant activation (560 – 585 ms).

Figure A7

Mean ECG Amplitude for Sex Groups in Total during Post-VH

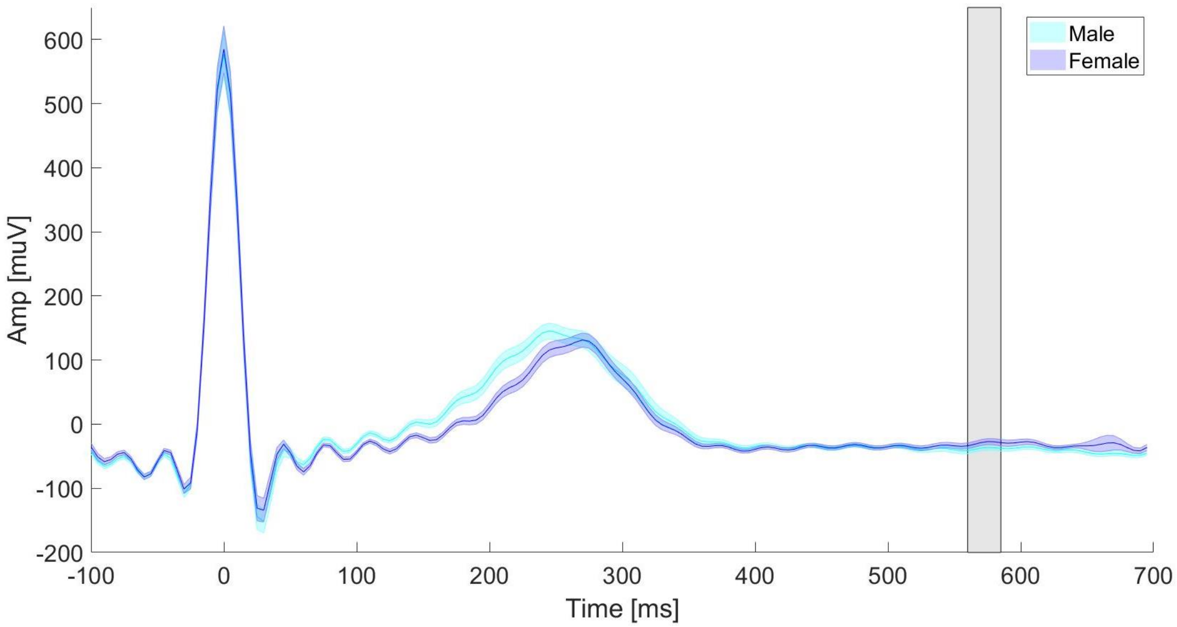

Note. Mean ECG amplitude averaged for each male and female participant within post-VH across epoch length. The gray shaded rectangle depicts the time period (560 – 585 ms) in which a significant positive electrode cluster was identified in the EEG (see Figure A5).
